# Stathmin Is Dispensable for Tumor Onset in Mice

**DOI:** 10.1371/journal.pone.0045561

**Published:** 2012-09-20

**Authors:** Sara D’Andrea, Stefania Berton, Ilenia Segatto, Linda Fabris, Vincenzo Canzonieri, Alfonso Colombatti, Andrea Vecchione, Barbara Belletti, Gustavo Baldassarre

**Affiliations:** 1 Division of Experimental Oncology 2, CRO, National Cancer Institute, Aviano, Italy; 2 Pathology Unit, CRO, National Cancer Institute, Aviano, Italy; 3 Dipartimento di Scienze e Tecnologie Biomediche and MATI Center of Excellence, University of Udine, Udine, Italy; 4 Division of Pathology, II University of Rome “La Sapienza”, Ospedale Santo Andrea, Rome, Italy; Virginia Commonwealth University, United States of Amercia

## Abstract

The microtubule-destabilizing protein stathmin is highly expressed in several types of tumor, thus deserving the name of oncoprotein 18. High levels of stathmin expression and/or activity favor the metastatic spreading and mark the most aggressive tumors, thus representing a realistic marker of poor prognosis. Stathmin is a downstream target of many signaling pathways, including Ras-MAPK, PI3K and p53, involved in both tumor onset and progression. We thus hypothesized that stathmin could also play a role during the early stages of tumorigenesis, an issue completely unexplored. In order to establish whether stathmin expression is necessary for tumor initiation, we challenged wild type (WT), stathmin heterozygous and stathmin knock-out (KO) mice with different carcinogens. Using well-defined mouse models of carcinogenesis of skin, bladder and muscle by the means of 7,12-dimethylbenz[α]antracene (DMBA)/12-O-tetradecanoylphorbol-13-acetate (TPA), N-butyl-N-(4-hydroxybutyl) nitrosamine (BBN) and 3-methylcholanthrylene (3MC) treatments, respectively, we demonstrated that knock-out of stathmin has no impact on the onset of cancer in mice. No significant difference was noticed either when the Ras oncogene was mutated (skin carcinogenesis model) or when the p53 pathway was inactivated (bladder carcinomas and fibrosarcomas). Finally, we concomitantly impinged on p53 and Ras pathways, by generating WT and stathmin KO mouse embryo fibroblasts transformed with papilloma virus large T antigen (LgTAg) plus the K-Ras^G12V^ oncogene. *In vivo* growth of xenografts from these transformed fibroblasts did not highlight any significant difference depending on the presence or absence of stathmin. Overall, our work demonstrates that stathmin expression is dispensable for tumor onset, at least in mice, thus making stathmin a virtually exclusive marker of aggressive disease and a promising therapeutic target for advanced cancers.

## Introduction

The phosphoprotein stathmin 1 (hereafter referred as stathmin) was initially identified as a cytosolic protein phosphorylated in response to several extracellular signals [1]. Further studies demonstrated that stathmin is a microtubule-destabilizing protein that is able to induce microtubules catastrophe [2] and to sequester free αß-tubulin heterodimers [3]. Stathmin N-terminal domain contains four serine residues (Ser16, Ser25, Ser38 and Ser63), which represent common phosphorylation targets of an expanding list of kinases, such as PI3K [4], [5], MAPK [6]–[9], PKA [10], [11], Calcium/Calmodulin kinase [12] and CDKs [10], [13]. Phosphorylation turns off the microtubule destabilizing activity of stathmin [14], is absolutely necessary for cells to enter mitosis, reviewed in [15] and modulates several fundamental cellular functions, such as cell motility, proliferation and apoptosis [16]–[22]. These observations are consistent with the name given to “stathmin”, deriving from the Greek word “stathmos” for “relay” to reflect its role as important intermediate of signal transduction.

Stathmin is overexpressed in several types of tumor, thus deserving its second name of oncoprotein 18 (OP18) [23]. In human cancers stathmin overexpression is associated with increased malignancy, metastasis formation and decreased patient overall survival [16], suggesting that stathmin could serve as a molecular marker to identify patients with more aggressive disease. This notion is in accord with the ability of stathmin to stimulate cell motility and invasion *in vitro* and metastasis formation in several models of human cancer [4], [20], [24]–[28]. Besides, high levels of stathmin has also been linked, both *in vitro* and *in vivo*, with increased cell proliferation and resistance to apoptosis [16] and its increased activity with aneuploidy and cell transformation [29], [30]. Stathmin is among the first identified genes repressed by the p53 wild type tumor suppressor gene [31], [32]. p53 regulation of stathmin expression represents an important regulatory event in the p53-dependent G_2_/M cell-cycle arrest [33]. Moreover, it has been demonstrated that stathmin is a target of and cooperates with mutant-p53 during liver cancer progression [34], [35]. Accordingly, knockdown of stathmin in mutant-p53 breast cancer cell lines decreases cell proliferation, viability and clonogenicity, partially restoring cell-cycle regulation and activation of apoptosis [36]. Overall, data from literature show that in both normal and cancer cells stathmin plays a pivotal role in mediating p53 signaling and also suggest that stathmin could play a role in cancer initiation. This hypothesis is corroborated by other evidences demonstrating that stathmin is necessary for the survival of p53-null cells [37] and is a direct target of PI3K and Ras-MAPK [4]–[9], two pathways frequently linked to tumor onset in humans.

However, while a large amount literature exists describing the role of stathmin in tumor progression, very little is known about its role during the early stages of tumorigenesis. We speculated that stathmin expression could be necessary for tumor initiation. To address this issue we compared the rate of tumor onset in wild type (WT), stathmin heterozygous and stathmin knock out (KO) mice [38] using well-defined mouse models of carcinogenesis. Moreover, we compared the tumorigenic potential of WT and stathmin KO mouse embryo fibroblasts transformed with papilloma virus large T antigen (LgTAg) plus the K-Ras^G12V^ oncogene. Unexpectedly, our data revealed that stathmin does not play any role in tumor onset in mice, reinforcing the hypothesis that stathmin could be used as marker of tumor aggressiveness and as therapeutic target in metastatic patients.

## Results

### Characterization of Stathmin Knock-out Mice

We previously demonstrated that stathmin plays a pivotal role in tumor dissemination by regulating cell motility, both *in vitro* and *in vivo* [20]–[22], [24], [25]. In order to investigate stathmin contribution in early steps of tumorigenesis we decided to compare the rate of tumor onset in WT and stathmin KO mice, using well-defined protocols of carcinogenesis.

To this aim, we first characterized stathmin expression in mouse tissues and evaluated effective knock-out of stathmin in KO animals. Due to the diverse sensitivity to the different carcinogens employed in our studies, we used two different mouse strains, namely the C57BL/6 and the FVB mice.

**Figure 1:**
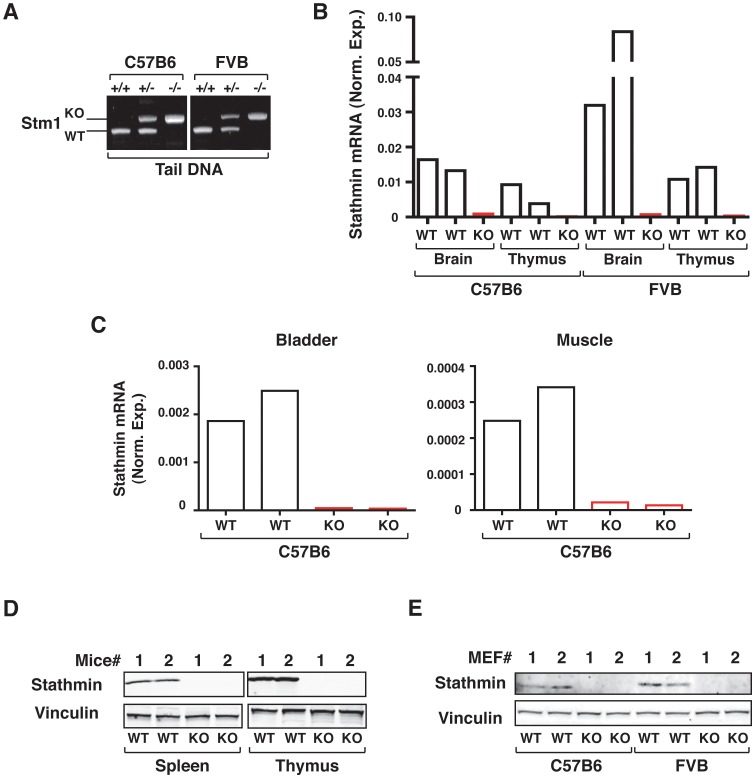
Stathmin is efficiently knocked out in C57BL/6 and FVB mouse strains. (A) PCR analyses of genomic DNA extracted from the tail of WT, stathmin heterozygous and KO C57BL/6 and FVB mice. (B) qRT-PCR analyses of stathmin mRNA expression in brain and thymus from 10 weeks old WT and stathmin KO C57BL/6 and FVB mice. (C) qRT-PCR analyses of stathmin mRNA expression in bladder and skeletal muscle from 10 weeks old WT and stathmin KO C57BL/6 mice. (D) and (E) Western Blot analysis of stathmin protein expression in thymus and spleen of 15 weeks old WT and stathmin KO C57BL/6 mice (D) and in mouse embryo fibroblasts (MEF) isolated from 13.5 days old C57BL/6 or FVB embryos (E). Vinculin was used as loading control.

Efficient stathmin KO was confirmed at genomic (Figure 1A), mRNA (Figure 1B–C) and protein (Figure 1D–E) levels, in both mouse organs (Figure 1B–D) and primary mouse embryonic fibroblasts (MEF) (Figure 1E). qRT-PCR analyses confirmed that stathmin mRNA is expressed in normal mouse organs, with highest levels detected in brain and thymus (Figure 1B–C), as expected [16]. In particular, the specific organs targeted by the carcinogens, the rear leg skeletal muscle and the bladder, expressed stathmin mRNA at detectable levels (Figure 1C). Organs from stathmin KO mice expressed undetectable levels of stathmin, at both mRNA and protein levels (Figure 1B–D).

Stathmin WT and KO mice were born at the expected Mendelian frequency and their growth and health were monitored up to one year of age. Although we did not study in depth the social behavior of stathmin null mice, we noticed that stathmin null females displayed some deficits in pup retrieval, especially for the care of the first litter, as previously reported [39]. This behavior was particularly evident in the FVB background, while not observable in stathmin heterozygous and WT females of both C57BL/6 and FVB strains (SDA and GB, personal observation). No other signs of different behavior and/or health condition were noticed, in accord with published data [38].

### Stathmin does not Affect Tumor Onset in p53-dependent Tumorigenesis

Since stathmin is a p53-repressed gene [31], [32] and is necessary for the survival of cells lacking the p53 tumor suppressor protein [37], we speculated that mouse models of p53-dependent tumorigenesis would be ideal to establish a role of stathmin in tumor initiation. Thus, we set up a fibrosarcoma model induced by the intramuscular injection of 3-methylcholanthrene (3MC) [40], [41] and a bladder carcinogenesis model, induced by treatment with N-butyl-N-(4-hydroxybutyl) nitrosamine (BBN) [41], [42]. Both in human sarcomas and bladder carcinomas stathmin is frequently upregulated in the early stages of tumor progression, as revealed by bioinformatic analyses using the Oncomine resource (Figure S1), suggesting that stathmin could be implicated in the onset of these types of cancer.

**Figure 2:**
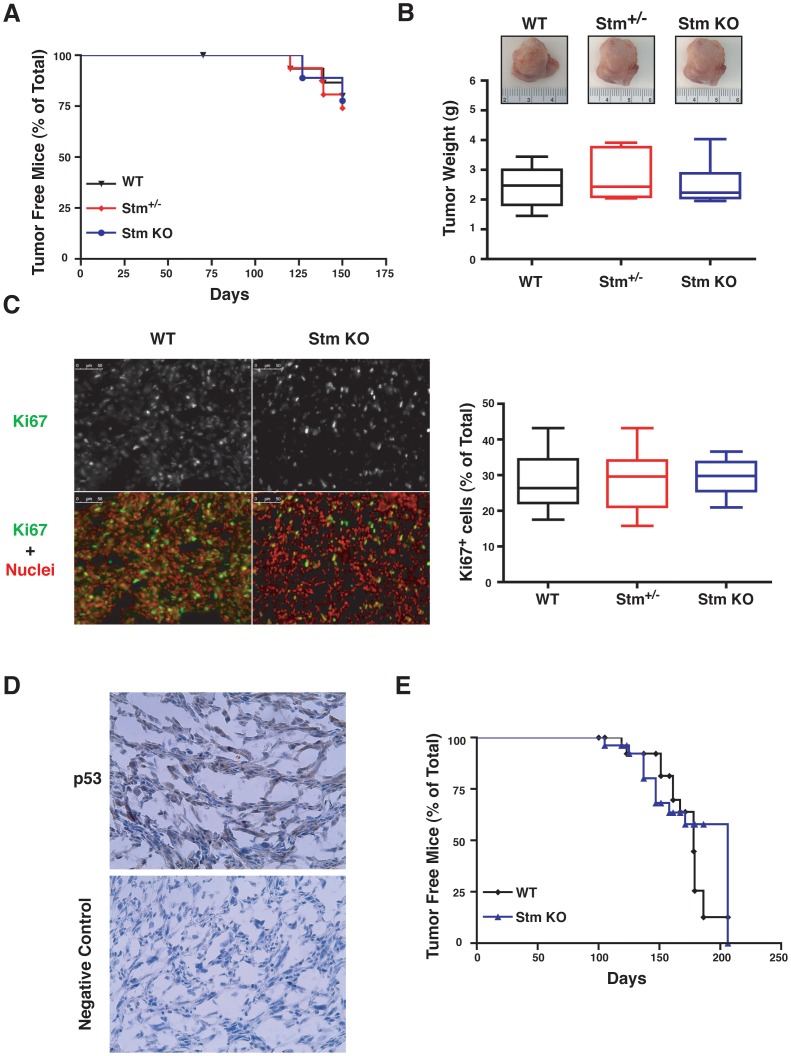
Stathmin is not required for tumor onset following treatment with 3MC in mice. (A) Kaplan Meier curves of tumor-free WT, stathmin heterozygous and KO mice challenged with 1 intramuscular injection of 3-methyl-cholanthrene (3MC). Mice were all sacrificed 150 days after treatment. *p = n.s.* using the Log Rank test. (B) Macroscopic appearance of tumor explanted from the mice treated as described in A. The box-plot reports the weight of tumors explanted 150 days after treatment. The horizontal bar in the box indicates the median weight in each genotype. (C) Typical immunofluorescence analysis of Ki67 expression in fibrosarcomas from WT and stathmin KO mice explanted 150 days after treatment. In the upper panels Ki67 staining is shown in gray. In the lower panels merging of Ki67 (green) and nuclei (red) staining is shown. On the right, the box-plot reports the quantification of Ki67 expression, expressed as percentage of positive cells over the total cell number/field. (D) Typical staining of p53 expression in fibrosarcomas, as evaluated by immunohistochemical analysis. The bottom panel shows the negative control, obtained processing the same samples without the primary anti-p53 antibody, demonstrating the specificity of the staining. (E) Kaplan Meier curves showing the number of tumor-free WT, stathmin heterozygous and KO mice challenged with 1 intramuscular injection of 3MC, using as end-point tumor appearance. *p = n.s.* using the Log Rank test.

First, we monitored the formation of fibrosarcomas in WT, stathmin heterozygous and KO C57BL/6 mice. Animals were sacrificed 150 days after 3MC injection and pathologically analyzed. Under these conditions comparable numbers of WT, stathmin heterozygous and KO mice developed macroscopically identifiable sarcomas (Figure 2A and Table 1). No significant difference was observed in tumor weight and size (Figure 2B) nor in local invasion or in the extent of necrosis (Table 1), between WT, stathmin heterozygous or KO mice. All tumors were diagnosed as spindle cell sarcomas showing, in some cases, areas of pleomorphism or round cell dedifferentiation (Table 1). Tumors displayed a similar proliferative rate, evaluated by Ki67 expression (Figure 2C) and showed no difference in p53 expression, as evaluated by immunohistochemistry (Figure 2D and Table 1) and western blot analyses (data not shown). Next, we followed the same protocol but used tumor appearance as endpoint. WT and stathmin KO mice were injected with 3MC and sacrificed when palpable tumors appeared. Also using this approach, we observed that no significant difference in tumor latency existed between WT and stathmin KO mice (Figure 2E).

**Table 1:** Histological characterization of 3-methyl-cholanthrene-induced sarcomas.

Genotype	Histotype^1^	Local Infiltration^2^	Necrosis^3^	Survival (Days)	p53^4^	p53 Localization
**WT**	Spindle + Round	0	1	139	ND	ND
**WT**	Spindle	1	0	120	0	
**WT**	Spindle	1	0	150	2	Nucleo + Cytoplasm
**Stm^+/−^**	Spindle + Pleomorphic	0	2	138	2	Cytoplasm
**Stm^+/−^**	Round	1	0	139	3	Nucleo + Cytoplasm
**Stm^+/−^**	Spindle	2	0	120	0	
**Stm KO**	Spindle	1	2	127	1	Cytoplasm
**Stm KO**	Spindle + Round	0	0	150	3	Nucleo +Cytoplasm

1 Tumors were classified according to their morphology as spindle, pleomorphic or round cell sarcomas. In some cases, multiple morphologic phenotypes were present in the same tumor.

2 Local Infiltration was rated as follows: 0  =  no tumor infiltration; 1  =  minimal tumor infiltration in the surrounding muscle; 2  =  massive tumor infiltration.

3 Necrosis was rated as follows: 0  =  no necrosis; 1  =  minimal areas of necrosis; 2  =  large areas of necrosis.

4 p53 staining was rated as follows: 0  =  no staining; 1  =  faint staining in less than 50% of the cells; 2  =  strong staining in less than 50% of the cells; 3  =  strong staining in more than 50% of the cells. ND  =  not done.

**Figure 3:**
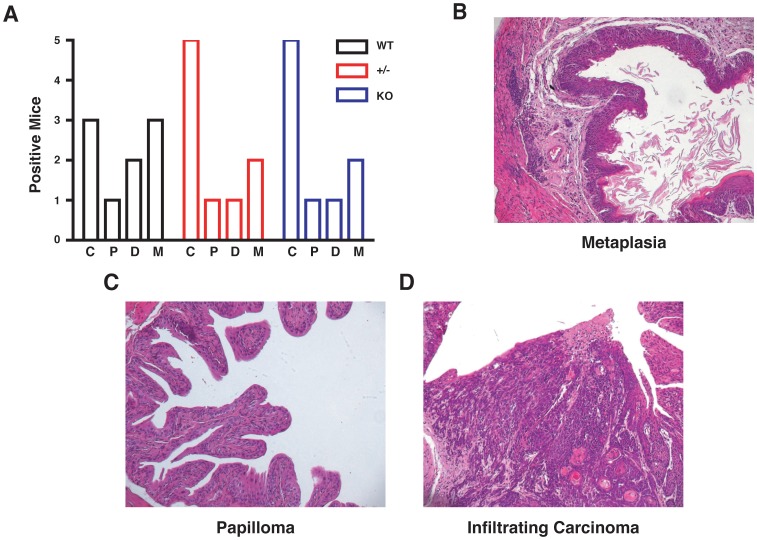
Stathmin is not required for tumor onset following treatment with BBN in mice. (A) Analysis of tumor appearance following treatment with N-butyl-N-(4-hydroxybutyl) nitrosamine (BBN) in mice of the indicated genotype. Mice were continuatively given BBN at 0,025% in the drinking water for 15 weeks, then left untreated for 3 additional weeks and sacrificed. Nine mice/genotype were used and bladder analyzed for the presence of tumoral tissue. In the graph, the number of events in each category is reported. C  =  Carcinoma; P  =  Papilloma; D  =  Dysplasia; M  =  Metaplasia. (B) Hematoxylin and Eosin staining of metaplasia, (C) papilloma and (D) invasive carcinoma in BBN-treated mice.

Next, we induced the formation of urinary bladder cancer, by treating C57BL/6 mice with the carcinogen BBN (9 mice/each genotype). Animals were sacrificed three weeks after the end of the BBN treatment. All bladders were collected, pathologically analyzed and classified for the presence of metaplasia, dysplasia, papillomas or carcinomas. Results from this analysis showed that in WT animals BBN treatment resulted in the appearance of 3 carcinomas and 1 papilloma while both stathmin heterozygous and KO mice displayed 5 bladder carcinomas and 1 papilloma (Figure 3A). Pathological analysis demonstrated that percentage of tumors displaying features of metaplasia (Figure 3B), papilloma (Figure 3C) or infiltrating carcinoma (Figure 3D) did not significantly change among the different genotypes. As expected, bladders from five age-matched untreated mice for each genotype showed no sign of pathological alterations (not shown).

Altogether, the results from these two *in vivo* approaches demonstrated that in p53-depedent carcinogenesis stathmin is dispensable for tumor onset in mice. Its absence somehow rather slightly increased the appearance of bladder carcinomas in the BBN model, but this increment was not significant and did not correlate with gene dosage, being present in both stathmin heterozygous and KO mice.

### Stathmin does not Affect Ras-driven Tumorigenesis

We next asked whether stathmin expression could be necessary for tumor onset in a model of tumorigenesis driven by the Ras oncogene. To this aim, we used the Ras-dependent skin carcinogenesis model, induced by treatment with 7,12-dimethylbenz[α]antracene (DMBA)/12-O-tetradecanoylphorbol-13-acetate (TPA) [43]. It is already known that stathmin expression is induced by DMBA treatment in mice [44] and also that stathmin is a downstream target of the Ras-MAPK pathway, supporting the hypothesis that it could play a role in this mouse model of carcinogenesis.

**Figure 4:**
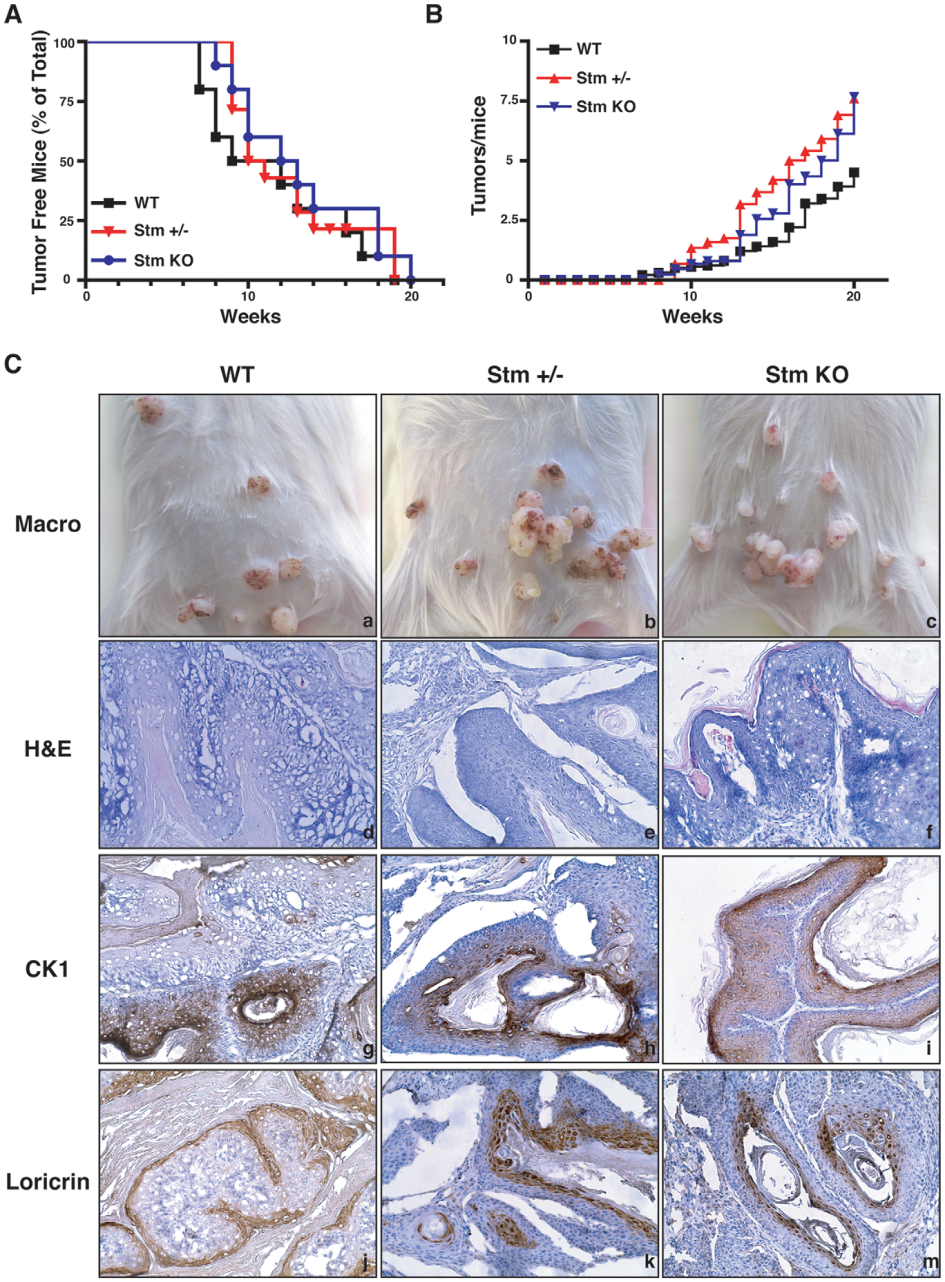
Stathmin is not required for tumor onset following DMBA/TPA treatment in mice. (A) Kaplan Meier curves of tumor-free WT, stathmin heterozygous and KO mice challenged with the 7,12-dimethylbenz[α]antracene (DMBA)/12-O-tetradecanoylphorbol-13-acetate (TPA) protocol, using as endpoint 20 weeks of treatment. p = n.s., using the Log Rank test. (B) Evaluation of the number of tumors/mice in function of time. *p = n.s.,* using the Mann Whitney test. (C) Macroscopic and microscopic analyses of papillomas in mice of the indicated genotypes. Typical images are reported. H&E  =  hematoxylin and eosin staining. CK1  =  IHC analysis of cytokeratin 1 expression. Loricrin  =  IHC analysis of loricrin expression.

Since C57BL/6 mouse strain is highly resistant to DMBA/TPA-induced carcinogenesis [43], we used for this experimentation the FVB mouse strain. Tumors started to appear after 7–8 weeks of TPA treatment with no significant difference in tumor latency among 10 WT, 12 heterozygous and 9 stathmin KO mice (Figure 4A). All mice developed tumors within 20 weeks (Figure 4A). Stathmin heterozygous and KO mice developed a slightly higher number of tumor/mouse respect to the WT ones, but the difference did not reach statistical significance (Figure 4B). The pathological and immunohistochemical analyses using loricrin and cytokeratins (CK) 1 and 8 as markers of tumor progression [43] revealed that all analyzed tumors were papillomas (*i.e.* loricrin and CK1 positive but CK8 negative) (Figure 4C). Thus, the absence of stathmin did not induce any change in the rate of papilloma-carcinoma conversion.

**Figure 5:**
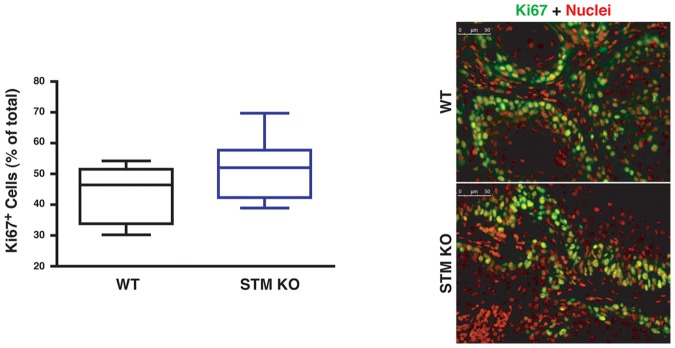
Analysis of tumor proliferation in WT and stathmin KO mice. On the left, the box-plot shows the percentage of Ki67 positive cells in tumors from WT and stathmin KO mice. *p = n.s.,* using the Mann Whitney test. On the right, typical images of Ki67 staining are reported, showing the merge of Ki67 (green) and nuclei (red) in tumors from WT and stathmin KO mice.

Tumors from WT and stathmin KO mice were also analyzed for their proliferative index, using Ki67 as marker of cell proliferation. As shown in Figure 5, tumors from stathmin KO animals showed a slight increase in the percentage of Ki67 positive cells, when compared to those from WT animals. However, also in this case the difference did not reach statistical significance (p = 0.06).

**Figure 6:**
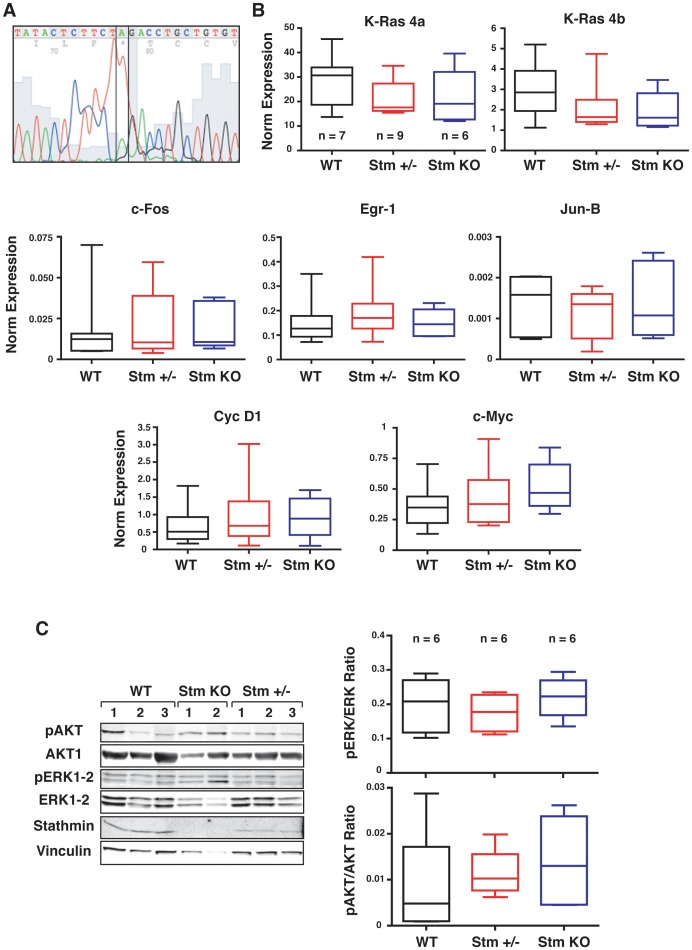
Molecular characterization of papillomas from WT, stathmin heterozygous and KO mice. (A) DNA sequence analysis, using the Sanger method, of H-Ras in tumors from WT, stathmin heterozygous and KO mice. The T-A substitution in heterozygosis is framed in black. A typical sequence is reported. (B) qRT-PCR analyses of K-Ras4a, K-Ras4b, c-Fos, Egr-1, Jun-B, Cyclin D1 and c-Myc mRNA expression in tumors from mice of the indicated genotype. The horizontal bar within the box indicates the median expression of each gene. (C) Western blot analyses of AKT and MAPK activation in papillomas derived from mice of the indicated genotype. On the right, the box-plot show the ratio between phosphorylated and total ERK (upper panel) and phosphorylated and total AKT (lower panel), in 6 tumors/genotype. *p = n.s.,* using the Mann Whitney test.

Moreover, the molecular characterization confirmed the lack of significant differences among the tumors from the WT, stathmin heterozygous and KO mice (Figure 6). All analyzed tumors showed the A-T (182) transversion in codon 61 of the H-Ras1 gene (Figure 6A), typical of the DMBA-induced carcinogenesis [43]. Independently from the genotype, similar levels of mRNA expression for K-Ras4A and 4B alternative splicing forms were observed, with the former expressed about 10-fold more than the latter, as evaluated by qRT-PCR analysis (Figure 6B). The mRNA of the transcription factors c-Fos, Egr-1 and Jun-B, downstream targets of the Ras-MAPK pathway, were also expressed at the similar levels in the tumors derived from WT, stathmin heterozygous and KO mice and the same was true for their transcriptional target cyclin D1 (Figure 6B). Finally, since it has been reported that the oncogene c-Myc could be involved in skin carcinogenesis initiation [45], we evaluated its expression in tumors from WT, stathmin heterozygous and KO mice. However, qRT-PCR analysis of c-Myc mRNA did not reveal significant differences in its expression level among the three genotypes (Figure 6B).

In accord with the results obtained from analysis of mRNA expression, the activation status of AKT and of ERK1/2 (analyzed by the expression of their phosphorylation on Ser 473 and on Thr 202/Tyr 204, respectively) was comparable in tumor lysates extracted from WT, stathmin heterozygous and KO mice (Figure 6C).Overall, our data demonstrated that reducing or ablating stathmin expression did not affect the number of tumors nor their latency nor the molecular alterations typical of the DMBA/TPA-induced carcinogenesis.

### Other Stathmin-family Members are not Overexpressed in Stathmin-1 KO Mice

**Figure 7:**
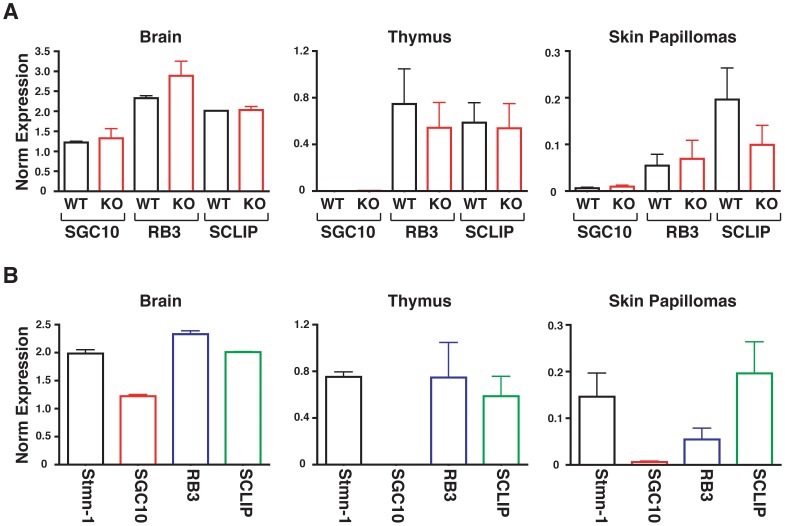
SCG10, SCLIP and RB3 are expressed at similar levels in WT and stathmin KO mice. (A) qRT-PCR analyses of the expression of SCG10, SCLIP and RB3 in brain, thymus and skin papilloma derived from WT and stathmin KO mice. (B) qRT-PCR analyses comparing the mRNA levels of stathmin 1, SCG10, SCLIP and RB3 in brain, thymus and skin papilloma from WT mice. Data represent the mean (±SD) of three mice/genotype (brain and thymus) or four mice/genotype (skin papillomas).

One possible explanation for the lack of effects of stathmin absence during mouse carcinogenesis could reside in compensatory overexpression of other members of the stathmin family, namely SCG10 (Superior Cervical Ganglia 10, also known as stathmin 2), SCLIP (SCG10-like protein, also known as stathmin 3) or RB3 (also known as stathmin 4) [46]. To establish whether this was the case in our model, we evaluated the mRNA expression of the other 3 members of the stathmin family in selected organs and tumors from WT and stathmin KO mice. In particular, we chose to analyze brains and thymuses since we previously observed that they displayed high levels of stathmin 1 in WT mice (Figure 1). Results from qRT-PCR analysis revealed that SCG10, SCLIP and RB3 were expressed at similar levels in RNA extracted from WT and stathmin KO mouse tissues and skin papillomas (Figure 7A). Analysis in WT mice revealed that expression of SCG10 was confined to the brain while SCLIP and RB3 were also expressed in thymus and in skin papillomas (at similar levels to stathmin-1) (Figure 7B). Together, these data indicated that KO of stathmin 1 did not result in increased expression of other members of the family. However, it is possible that during tumor development SCLIP and, with lesser extent, RB3 could compensate for the loss of stathmin 1 activity in the stathmin KO mice.

### Stathmin is Dispensable for Ras-induced Transformation

**Figure 8:**
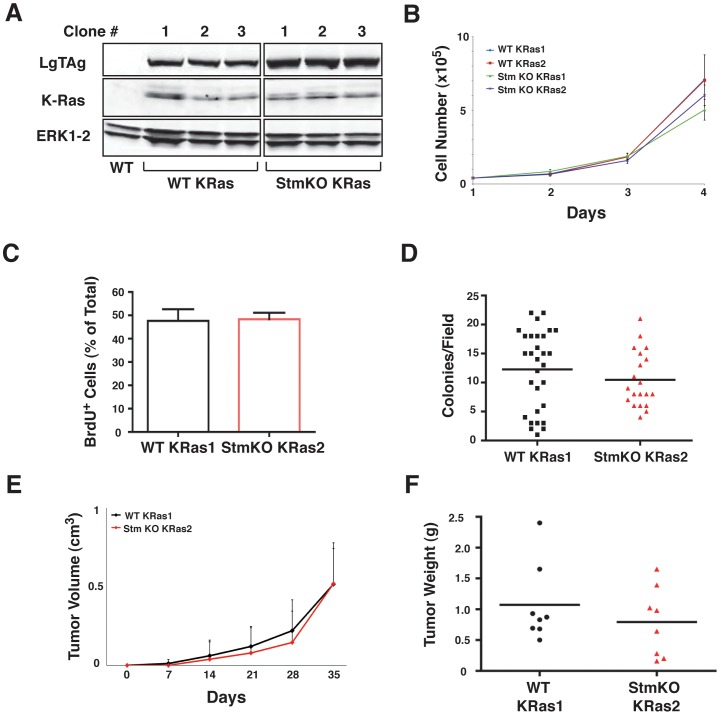
Tumorigenic potential of MEF transformed with LgTAg and K-Ras4b^G12V^ does not depend on stathmin expression. (A) Western blot analysis showing the expression of papilloma virus Large T Antigen (LgTAg) (upper panel), K-Ras (middle panel) and ERK1/2 (lower panel) in mouse embryo fibroblasts (MEF) of the indicated genotype, transduced with retroviruses encoding for LgTAg and K-Ras4b^G12V^. In the first lane (WT), the protein lysate from non-transduced WT MEF was used as negative control for K-Ras and LgTAg expression. (B) Growth curve analysis on two independent K-Ras clones/genotype. Data represent the mean of 3 independent experiments performed in duplicate. (C) Graph reports the analysis of BrdU incorporation in the indicated cell clones, exposed to BrdU for two hours and then fixed and analyzed by immunofluorescence. Data are expressed as percentage of BrdU positive cells respect to the total and represent the mean of 3 independent experiments in which at least 200 cells were counted. (D) Graph reports results from soft agar assay. The numbers of colonies/field (using a 10 × objective) formed by the indicated cell clones in two independent experiments are reported. The black bar indicates the median number of colonies for each cell clone. (E) *In vivo* growth of WT and stathmin KO MEF transformed with LgTAg + K-Ras4b^G12V^, evaluated by measuring tumor volume each week for 5 weeks in 8 mice/genotype. *p = n.s.,* using the Mann Whitney test. (F) Graph reports the weight of the tumors explanted from mice injected with LgTAg + K-Ras4b^G12V^ transformed WT and stathmin KO MEF. The black bar indicates the median tumor weight. *p = n.s.,* using the Mann Whitney test.

We then speculated that the expression of stathmin might be important in initiation of tumors in which concomitant alteration of the p53 and Ras-MAPK pathways are present. To this aim, we used an *in vitro* model based on the expression of papilloma virus Large T Antigen (LgTAg) plus the K-Ras^G12V^ oncogene in mouse embryo fibroblasts (MEF). In this way, the process of cellular transformation impinges concomitantly on the p53 and the Ras-MAPK pathways. WT and stathmin KO MEF were transduced with LgTAg plus the K-Ras^G12V^ oncogene (Figure 8A) and analyzed for their proliferation and transformed phenotype. The analyses of cell proliferation using the growth curves (Figure 8B) and the BrdU incorporation assay (Figure 8C) demonstrated that stathmin null transformed cells proliferate at the same extent of their WT counterpart. Similarly, no significant difference was observed in their anchorage independent growth (Figure 8D).

To better understand whether the absence of stathmin could have an effect in the tumorigenic potential of these transformed cells, we decided to evaluate their growth *in vivo*. To this aim, we subcutaneously injected cells in nude mice and monitored for tumor appearance and development over the next 5 weeks. Also in this case we did not observe any significant difference in tumor latency and growth, evaluated both as tumor volume (Figure 8E) and tumor mass (Figure 8F).

Taken together our studies demonstrated that stathmin expression is dispensable for the p53- and Ras-dependent carcinogenesis, at least in mice.

## Discussion

Here, we address the role of stathmin in mouse tumorigenesis using well-defined models of carcinogenesis. Overall, our data demonstrate that stathmin absence does not influence the onset of bladder and skin carcinomas or fibrosarcomas in mice, suggesting that stathmin is dispensable for initiation of p53- and Ras-dependent tumors in mice. Our results also excluded that, at least in mice, stathmin knock-down is balanced by the increased expression of other members of the stathmin family, since they were not differentially expressed in WT *vs* stathmin KO mouse organs. However, based on our results from analysis of skin papillomas, we cannot exclude that SCLIP and RB3 can compensate for the absence of stathmin 1, eventually allowing for tumor onset.

Taking into account the large literature regarding the role of stathmin in tumors, in cell proliferation and survival, in both normal and transformed cells, our data are quite surprising. Our data using the p53-dependent tumorigenesis models (i.e. BBN and 3MC) suggested that the absence of stathmin rather stimulated, more than inhibited, tumor formation, even though the differences were not statistically significant. More extensive studies will be necessary to better address this possibility. The *in vivo* data collected using the models of carcinogenesis were then corroborated by the use of WT and stathmin KO MEFs transformed with LgTAg and K-Ras^G12V^. No significant difference depending on the presence/absence of stathmin was highlighted, both *in vitro* and *in vivo*. This is somehow unexpected especially in the p53-dependent tumorigenesis model taking into account that stathmin is necessary for the survival of p53-null [37] and p53-mutated cells [36] and that human sarcomas and bladder carcinomas (Figure S1) frequently overexpress stathmin in the early stages of tumorigenesis. In light of the present results it is possible to speculate that these increased levels do not represent a *conditio* for tumor onset but rather a consequence of the fact that stathmin expression is often upregulated in highly proliferating tissues [47]–[49].

Current literature indicates that stathmin is frequently overexpressed in advanced stages of tumor progression, suggesting that stathmin could play a pivotal role during invasion and metastasis formation, in several types of cancer [4], [20], [24]–[28], [50]–[51]. Accordingly, in many human cancers high levels of stathmin have been proposed as a marker to identify patients with worse prognosis [16], [52]. In light of these observations, it would be interesting to clarify in the future whether overexpression of stathmin is able to worsen the tumor phenotype, using transgenic or knock-in mouse models in which the WT protein or its “pro-oncogenic” mutant forms [24], [29], [30] are introduced. Moreover, crossing the stathmin KO mice with mouse model prone to develop metastatic cancer, such as the p53^R172H^ knock-in mouse [53], could be particularly illuminating to fully comprehend stathmin role in tumor dissemination.

Although we report negative results regarding the role of stathmin in tumor onset, we believe that our work is of particular relevance and it will help to better delineate the potential applications of an “anti-stathmin” therapy in human cancer. shRNAs [54], ribozime [17], [55] or small molecules [56] designed to counteract stathmin expression and/or activity could represent important tools in the next future but, in light of our findings, they should probably be used to target highly aggressive metastatic tumors. The fact that stathmin expression is usually low in normal tissues allows the prediction that unwanted side effects using this type of therapy would be minimal. Stathmin null mice are generally healthy and do not develop major pathologies [38], [39] indirectly confirming the hypothesis that an “anti-stathmin” therapy would be well tolerated. In addition to that, the evaluation of stathmin expression and/or of its phosphorylation levels could also represent valid prognostic markers of tumor aggressiveness, at least in some types of human cancer. However, the possibility that an organ represents an exception to this rule should be seriously taken into account, as recently demonstrated for pelvic serous carcinomas [57].

In conclusion, using different types of mouse carcinogenesis models we report that stathmin is dispensable for tumor onset, at least in mice. Nevertheless, stathmin overexpression is likely to play an important role in tumor progression and future studies, focused on its significance in late stages of tumorigenesis, will probably define stathmin role as novel therapeutic target in advanced disease.

## Materials and Methods

### Mice

Animals used for experimentation received humane care and all *in vivo* experiments were performed in accordance with institutional regulations. Mice were housed under pathogen free conditions and under a 12-hour dark/light cycle. All procedures for animal experiments were approved by the Ethical Committee for Animal Experimentation (CESA) and performed in accordance with the institution guidelines. C57BL/6 stathmin KO mice (kindly provided by Dr. U.K. Schubart, NY, USA and Dr. M. Kuhn, Germany) [20], [38] were crossed with C57BL/6 WT animals (Charles River) to obtain stathmin heterozygous mice. Stathmin KO mice were born at the expected Mendelian frequency and did not show any evident sign of disease, in accord with published data [38]. For skin carcinogenesis, C57BL/6 stathmin KO mice were crossed with FVB WT mice (Charles River) until N6, to obtain near congenic (98.4% pure) FVB strain. All mice were monitored twice a week and euthanized when required, in accordance to the “AVMA guidelines on Euthanasia”.

### Genomic DNA Extraction and Genotyping

Genomic DNA extraction from mouse tail was performed using Maxwell 16 Mouse Tail DNA Purification kit (Promega, used with the Maxwell® 16 SEV Instrument). Genotype was determined by PCR using the following primers for the stathmin gene: OP18 WT (forward) 5′-GAGAATCCATGATTGCCAGCA-3′, OP18 KO (forward) 5′-CTAATGGCTATAGTTTCATGTTCC-3′ and OP18 REV (reverse) 5′-AGCAAAACCAAATTAAGGGCCAGC-3′.

### Carcinogenesis

For the induction of fibrosarcomas, 3–5 months old C57BL/6 mice received a single intra-muscular injection of 40 µl solution containing 3-methyl-cholanthrene (3MC) (Sigma-Aldrich) in one of the rear legs. 3MC was used at a concentration of 25 µg/µl and dissolved in sesame oil, accordingly to published procedures [40], [41]. Mice were monitored regularly and sacrificed 150 days after injection or when tumors reached approximately 1.5 cm in diameter.

For the induction of urinary bladder carcinomas, 3–5 months old C57BL/6 mice were exposed to N-butyl-N-(4-hydroxybutyl) nitrosamine (BBN, TCI, Japan) permanently present in the drinking water at a concentration of 0.025%, for 15 weeks and then left untreated for 3 additional weeks, as previously described with minor modifications [41], [42]. Mice were then sacrificed and the urinary bladders were pathologically analyzed.

For skin carcinogenesis, 7–9 weeks old FVB mice (10 WT, 12 stathmin heterozygous and 9 KO) were treated once with 25 µg of 7,12-dimethylbenz[α]anthracene (DMBA, Sigma-Aldrich) in acetone and, 2 weeks later, with 5µg of 12-O-tetradecanoylphorbol-13- acetate (TPA, Sigma-Aldrich) in acetone for 20 weeks (once a week) [43]. Animals were monitored twice/week and papillomas were counted and measured once a week. All mice were sacrificed two weeks after last application of TPA and tumors were explanted and analyzed.

### Immunohistochemistry

For immunohistochemistry, tumors were fixed in formalin over night (ON) at 4°C, embedded in OCT medium and then sectioned with a cryostat. After washes in PBS, tissue sections of 5 µm were immersed into 10 mM citrate buffer pH 6.0 and antigens were retrieved by boiling in microwave (550 W, 20 minutes). Samples were slowly cooled down to room temperature (RT), treated with H_2_O_2_ for 5 minutes to block endogenous peroxidase activity, permeabilized 5 minutes with 0.5% Triton X-100 and then blocked 1 hour in 10% normal goat serum at RT. Incubation with primary antibodies (anti-p53, Novocastra; anti-loricrin, COVANCE; anti-cytokeratin 1, COVANCE, anti-cytokeratin 8, AbCam) was performed ON at 4°C. Secondary anti-rabbit HRP-conjugated antibody was incubated for one hour at RT and developed with peroxidase substrate kit (VECTOR) for appropriate times. Section were then counterstained with hematoxylin, dehydrated in ascending alcohol series and cover-slipped.

### Cell Culture

Primary mouse embryonic fibroblasts (MEF) were isolated from WT and stathmin KO 13.5 days old embryos, from both C57BL/6 and FVB mouse genetic backgrounds. MEFs were cultured in DMEM (Lonza) supplemented with 10% fetal bovine serum (Sigma-Aldrich) according to standard procedures, as previously described [20].

To generate stable transformed cell clones of MEFs, primary cultures of fibroblasts at passage 3 have been used. Cells have been concomitantly transduced with SV40 Large T Ag (gently provided by Dr. R. Maestro, Centro di Riferimento Oncologico, Aviano, Italy, as BamH1 insert, and then cloned in pMSCV-puro retroviral vector, Clontech) and K-Ras4B^V12^ (obtained by the ADDGENE consortium and then cloned in pMSCV-hygro retroviral vector, Clontech). Cell clones and/or pools were selected with 1.5 µg/ml Puromycin and 0.4 mg/ml Hygromycin. The stable expression of the different constructs was tested by Western Blot analysis of the target proteins. Each experiment has been performed using at least two independent clones or pools.

### Proliferation Assays

For growth curves, 1×10^5^ cells/well were seeded in 6-well plates in complete medium in triplicate. Medium was replaced twice a week. At the indicated times, cells were detached using trypsin-EDTA (Lonza) and counted by Trypan Blue exclusion test.

For Ki67 staining, tissues treated as for IHC, were incubated ON at RT with rabbit anti-Ki67 antiserum (Abcam, Cambridge, UK). Then, incubation with anti-rabbit AlexaFluor488-conjugate (Invitrogen) and with propidium iodide 3 µg/ml + RNase A 100 µg/ml for 1 hour at RT was performed. Images were acquired using Leica fluorescence microscope (DMI6000B) equipped with a 40 × objective.

For BrdU incorporation assay, MEFs were seeded in 12-well plates (BD, Falcon) containing cover slips (Menzel-Glaser, 12 mm) and were incubated in standard growth conditions for 2 hour with 10 µM BrdU (Roche). Then, cells were fixed 20 minutes in 4% paraformaldehyde (PFA) at room temperature (RT) and permeabilized 30 minutes in HCl 1,5 N at 37°C. Cover slips were washed 2 times in Borate Buffer 0.1 M pH 8.5 and 2 times in PBS. Incubation with primary antibody anti-BrdU (Roche) was performed 1 hour at 37°C, then samples were washed in 3 times PBS and incubated with anti-mouse Alexa Fluor488-conjugate (Invitrogen) for 30 minutes at 37°C. Finally, nuclear staining with propidium iodide 3 µg/ml + RNase 100 µg/ml for 30 minutes at RT was performed and cover slips were mounted on glass slides using Mowiol 4–88 (Calbiochem).

To evaluate the anchorage-independent cell growth, K-Ras^V12^ transformed cells (5 × 10^3^) were resuspended in 0.4% agar (Low Melting Agarose, Sigma-Aldrich) in complete medium and quickly overlaid on a previously gelified 0.6% bottom agar (Low Melting Agarose, Sigma-Aldrich). The assay was performed in 6-well tissue culture plates, in triplicate. Fresh medium was added to the wells twice a week. On day 15, the number of colonies was counted in 10 random fields, using 10 × objective (Nikon Eclipse TS100).

### Western Blot Analysis

To extract total proteins from tumor specimens, tissues were disrupted using the TissueLyser II (QIAGEN) and lysed in NP40 lysis buffer (0.5% NP40; 50 mM HEPES pH 7; 250 mM NaCl; 5 mM EDTA; 0.5 mM EGTA, pH 8) containing a protease inhibitor cocktail (Complete™, Roche) and supplemented with 1 mM Na_3_VO_4_ (Sigma-Aldrich), 10 mM NaF (Sigma-Aldrich) and 1 mM DTT (Sigma-Aldrich). For immunoblotting analysis, proteins were separated in 4–20% SDS-PAGE (Criterion Precast Gel, Bio-Rad) and transferred to nitrocellulose membranes (GE, Healthcare). Membranes were blocked in 5% non fat dried milk in TBS-0.1% Tween20 or in Odyssey Blocking Buffer (Licor, Biosciences) and incubated at 4°C overnight with primary antibodies. Antibodies used for immunoblotting were: Stathmin (Sigma-Aldrich), vinculin (Santa Cruz), p53 (Novocastra), pERK (Cell signaling), ERK (Santa Cruz), pAKT (Cell Signaling), AKT (Santa Cruz), K-Ras (Santa Cruz) and LgTAg (Santa Cruz).

### Reverse Transcriptase-polymerase Chain Reaction and Quantitative Real-time PCR

Total RNA from mouse tumor samples was extracted with TRIzol (Invitrogen). Disruption of the tissue sample was achieved with the TissueLyser II (QIAGEN). Complete homogenization was achieved by passing the lysate at least 5 times through a 23-gauge needle fitted to an RNAse-free syringe. RNA was then quantified and retro-transcribed with AMV Reverse transcriptase (according to provider’s instruction, Promega) to obtain cDNAs. Absolute expression of mouse cyclin D1, c-Fos, stathmin, mouse Egr-1, Jun-B, K-Ras-4a and K-Ras-4b and c-Myc were evaluated by qRT-PCR, using EvaGreen dye-containing reaction buffer (SoFast EvaGreen Supermix, Bio-Rad in a MyiQ2 icycler, Bio-Rad).

The following primers (Sigma-Aldrich) were used:

mouse STM1 FW: 5′-GTTCGACATGGCATCTTCTGAT-3′

mouse STM1 Rev 5′-CTCAAAAGCCTGGCCTGAA-3′

mouse PGK1 FW: 5′-TACCTGCTGGCTGGATGG-3′

mouse PGK1 Rev: 5′-CACAGCCTCGGCATATTTCT-3′

mouse GusB FW: 5′-CTCTGGTGGCCTTACCTGAT-3′

mouse GusB Rev:5′-CAGTTGTTGTCACCTTCACCTC-3′

mouse JUN-b FW: 5′-GCACTAAAATGGAACAGCCCTT-3′

mouse JUN-b Rev: 5′-GGCTCGGTTTCAGGAGTTTG-3′

mouse EGR-1 FW: 5′-CCTTCCAGTGTCCAATCTGCA-3′

mouse EGR-1 Rev: 5′-CTGGCAAACTTCCTCCCACA-3′

mouse c-FOS FW: 5′-TGGTGAAGACCGTGTCAGGA-3′

mouse c-FOS Rev: 5′-GCAGCCATCTTATTCCGTTCC-3′

mouse cyclin D1 FW: 5′-TGGCCT CTAAGATGAAGGAGA-3′

mouse cyclin D1 Rev 5′-AGGAAGTGTTCGATGAAATCGT-3′

mouse k-Ras 4a FW: 5′CCTGGTAGGGAATAAGTGTGATTTG-3′

mouse k-Ras 4a Rev: 5′-GTACTGTCGGATCTCTCTCACCAAT-3′

mouse k-Ras 4b FW: 5′-GAGTAAAGGACTCTGAAGATGTGCC-3′

mouse k-Ras 4b Rev: 5′-CATCGTCAACACCCTGTCTTGTCTT-3′

mouse c-myc FW: 5′-TCTTTCCCTACCCGCTCAAC-3′

mouse c-myc Rev: 5′-ACCCTGCCACTGTCCAACTT-3′

mouse stathmin1 FW: 5′- GACCCACAAAATGGAGGCTA-3′

mouse stathmin1 Rev: 5′- TCTTCCACGTGCTTGTCCTT-3′

mouse SCG10 FW: 5′- GCGTGCACATCCCTACAATG -3′

mouse SCG10 Rev: 5′- CCCGCTTGTTGATCTGCTTC -3′

mouse SCLIP FW: 5′- AGGAGTTATCTGTGCTGTCGC -3′

mouse SCLIP Rev: 5′- TGGTAGATGGTGTTCGGGTG -3′

mouse RB3 FW: 5′- CCTGAACTGGTGCGTCATCT -3′

mouse RB3 Rev: 5′- TTAAACTCAGGCACCCCGTC -3′

Standard curves (10-fold dilution from 10^1^ to 10^−4^ attomoles) were prepared for both target genes and housekeeping genes. The incorporation of the SYBR Green dye into the PCR products was monitored in real time using the MyiQ2 iCycler (Bio-Rad), and the resulting threshold cycles (Ct) were computed. Ct values were converted into attomoles and the normalized target gene value was obtained by using two different housekeeping genes (GusB and PGK).

### H-Ras Sequencing

H-Ras1 was amplified from tumors’ RNA using the following primers: H-Ras-FW: 5′-GATTGGCAGCCGCTGTAG-3′ and H-Ras-Rev: 5′-CCATTGGCACATCATCTGAA-3′ and then sequenced with primer 5′-GTACTGATGGATGTCCTCGAA-3′, using BygDye® Terminator v3.1 Cycle Sequencing kit (Applied Biosystems).

### Statistics

All variables were expressed as mean±standard deviation (SD). Data were examined using the two-tailed Student t test or unpaired two-tailed Mann-Whitney U test. Differences were considered significant at p<0.05. Significance in tumor-free survival curves was calculated using Log Rank test. The computer software PRISM (version 4, GraphPad, Inc.) was used to make graphs and all statistical analyses.

### Ethics Statement

This study was carried out in strict accordance with the recommendations contained in the Guide for the Care and Use of Laboratory Animals of the Centro di Riferimento Oncologico, Natl. Cancer Institute, Aviano Italy. The project was approved by the Committee for the Ethics of Animal Experiments (CESA) of the Centro di Riferimento Oncologico (within the AIRC investigator grant # 8551). All mice were monitored twice a week and euthanized when required in accordance to the “AVMA guidelines on Euthanasia”. All efforts were made to minimize suffering.

## Supporting Information

Figure S1 **Stathmin is overexpressed in human Bladder Carcinomas and Sarcomas.** (A) Oncomine bioinformatic analyses of stathmin expression in bladder carcinomas in the Dyrskjot Dataset. Stathmin is overexpressed in the first stages of bladder carcinogenesis (stage 0 is, left panel), in superficial cancer (middle panel) and infiltrating carcinomas (right panel). (B) Stathmin expression in bladder carcinomas in the Sanchez-Carbayo Dataset, showing upregulation in infiltrating carcinomas respect to normal bladder. (C) Stathmin expression in human sarcomas in the Detwiller Dataset. Stathmin expression levels in fibrosarcomas (left panel) and in pleomorphic liposarcomas (right panel) are compared to those of normal tissues. With the exception of brain, both types of sarcomas display significant upregulation of stathmin respect to normal tissues. (D) Stathmin expression in human liposarcomas in the Barretina Dataset, showing upregulation of stathmin mRNA respect to normal adipose tissue.
(PDF)
