# Supplementary figures and images for: Stathmin Is Dispensable for Tumor Onset in Mice
Source: PLoS One. 2012 Sep 20;7(9):e45561. doi: 10.1371/journal.pone.0045561 (PMC3447788; doi:10.1371/journal.pone.0045561)

A

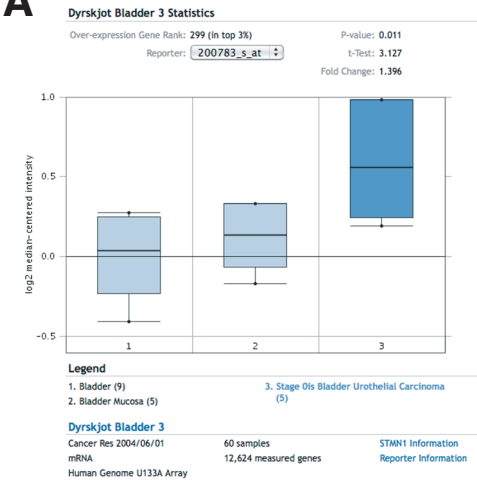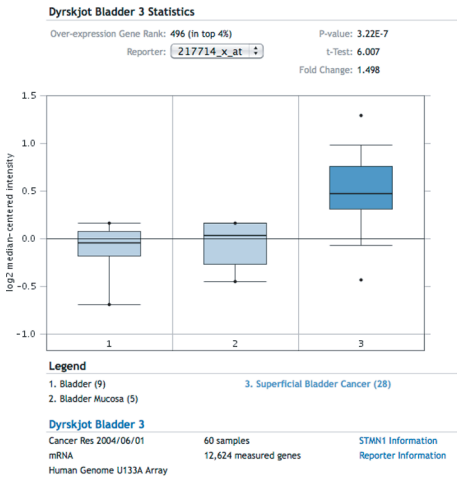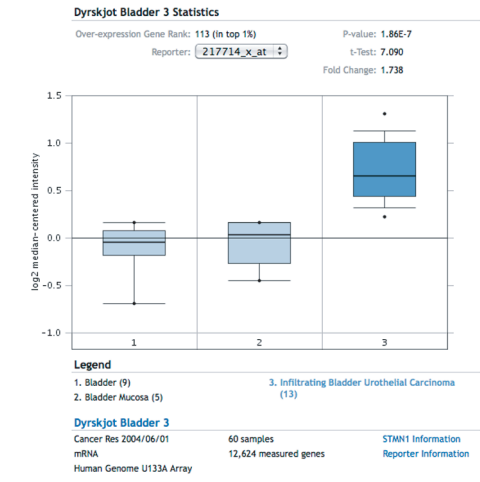

B

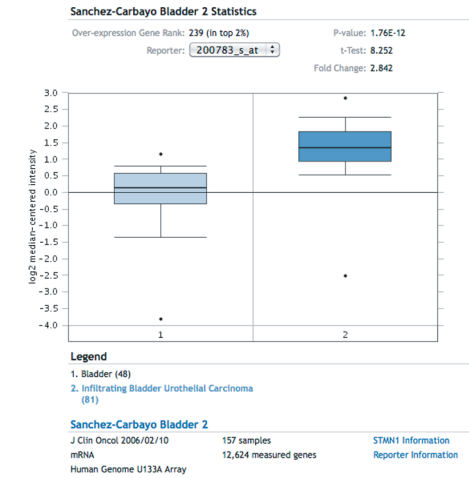

C

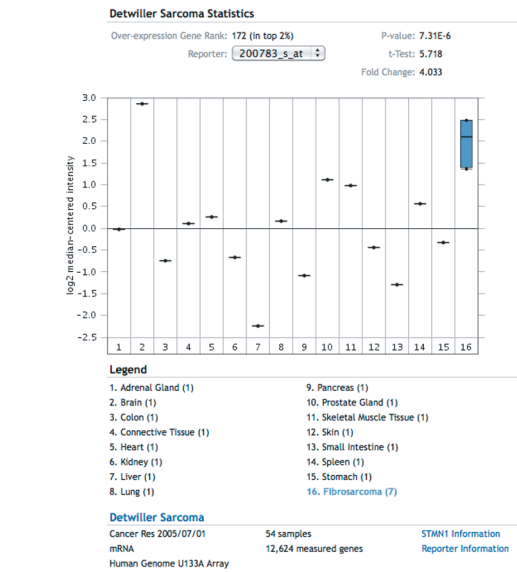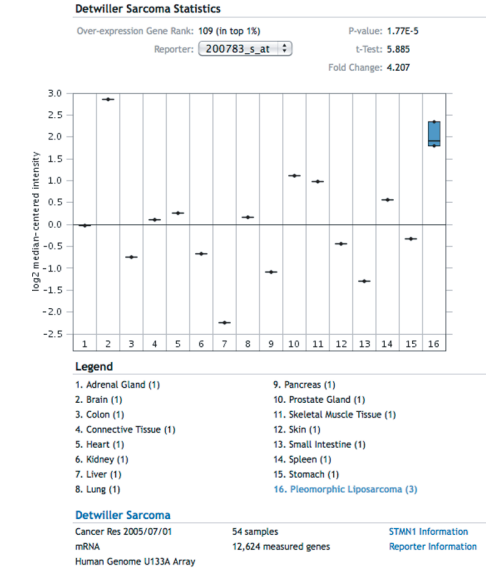

D

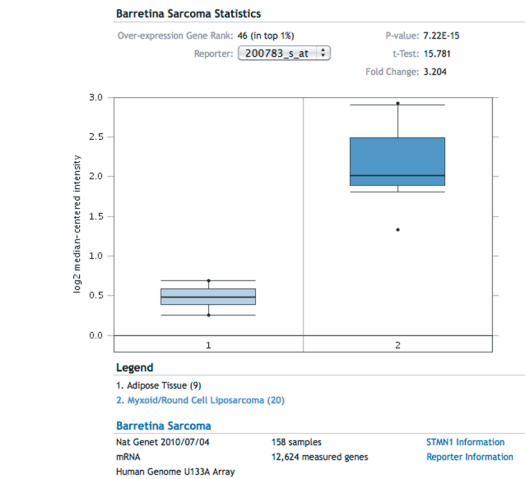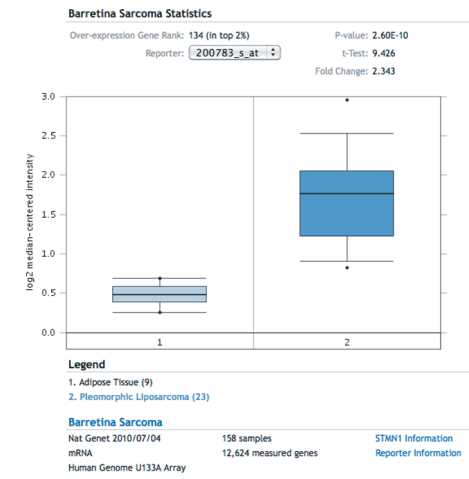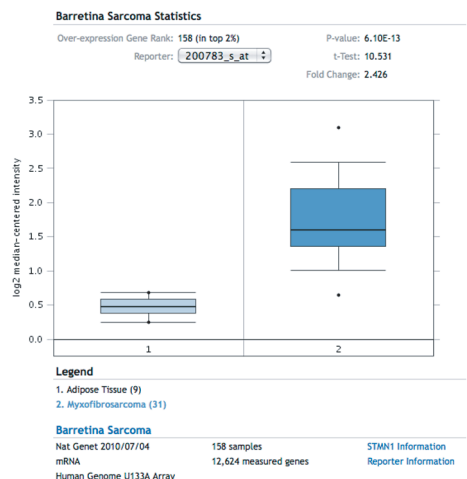

Supplement: Figure S1 — Stathmin is overexpressed in human Bladder Carcinomas and Sarcomas. (A) Oncomine bioinformatic analyses of stathmin expression in bladder carcinomas in the Dyrskjot Dataset. Stathmin is overexpressed in the first stages of bladder carcinogenesis (stage 0 is, left panel), in superficial cancer (middle panel) and infiltrating carcinomas (right panel). (B) Stathmin expression in bladder carcinomas in the Sanchez-Carbayo Dataset, showing upregulation in infiltrating carcinomas respect to normal bladder. (C) Stathmin expression in human sarcomas in the Detwiller Dataset. Stathmin expression levels in fibrosarcomas (left panel) and in pleomorphic liposarcomas (right panel) are compared to those of normal tissues. With the exception of brain, both types of sarcomas display significant upregulation of stathmin respect to normal tissues. (D) Stathmin expression in human liposarcomas in the Barretina Dataset, showing upregulation of stathmin mRNA respect to normal adipose tissue. (PDF) [file pone.0045561.s001.pdf]
